# Supplementary material for: Evaluation of Anthocyanin Profiles in Various Blackcurrant Cultivars over a Three-Year Period Using a Fast HPLC-DAD Method
Source: Foods. 2021 Jul 29;10(8):1745. doi: 10.3390/foods10081745 (PMC8394496; doi:10.3390/foods10081745)
Supplement: Supplementary file 1 [file foods-10-01745-s001.zip › foods-1300412-supplementary.pdf]

Evaluation of anthocyanin profiles in various blackcurrant cultivars over a three-years period using fast HPLC-DAD method

Barbora Šimerdová [1], Michaela Bobríková [1], Ivona Lhotská [1], Jiří Kaplan [2], Alena Křenová [2], Dalibor Šatínský [1*]

[1] The Department of Analytical Chemistry, Faculty of Pharmacy in Hradec Králové, Charles University, Czech Republic
[2] The Research and Breeding Institute of Pomology Holovousy, Holovousy 129, Hořice 508 01, Czech Republic
* Corresponding author: satinsky@faf.cuni.cz, tel. +420495067228

Supplementary material

1. HPLC analysis of blackcurrant cultivars

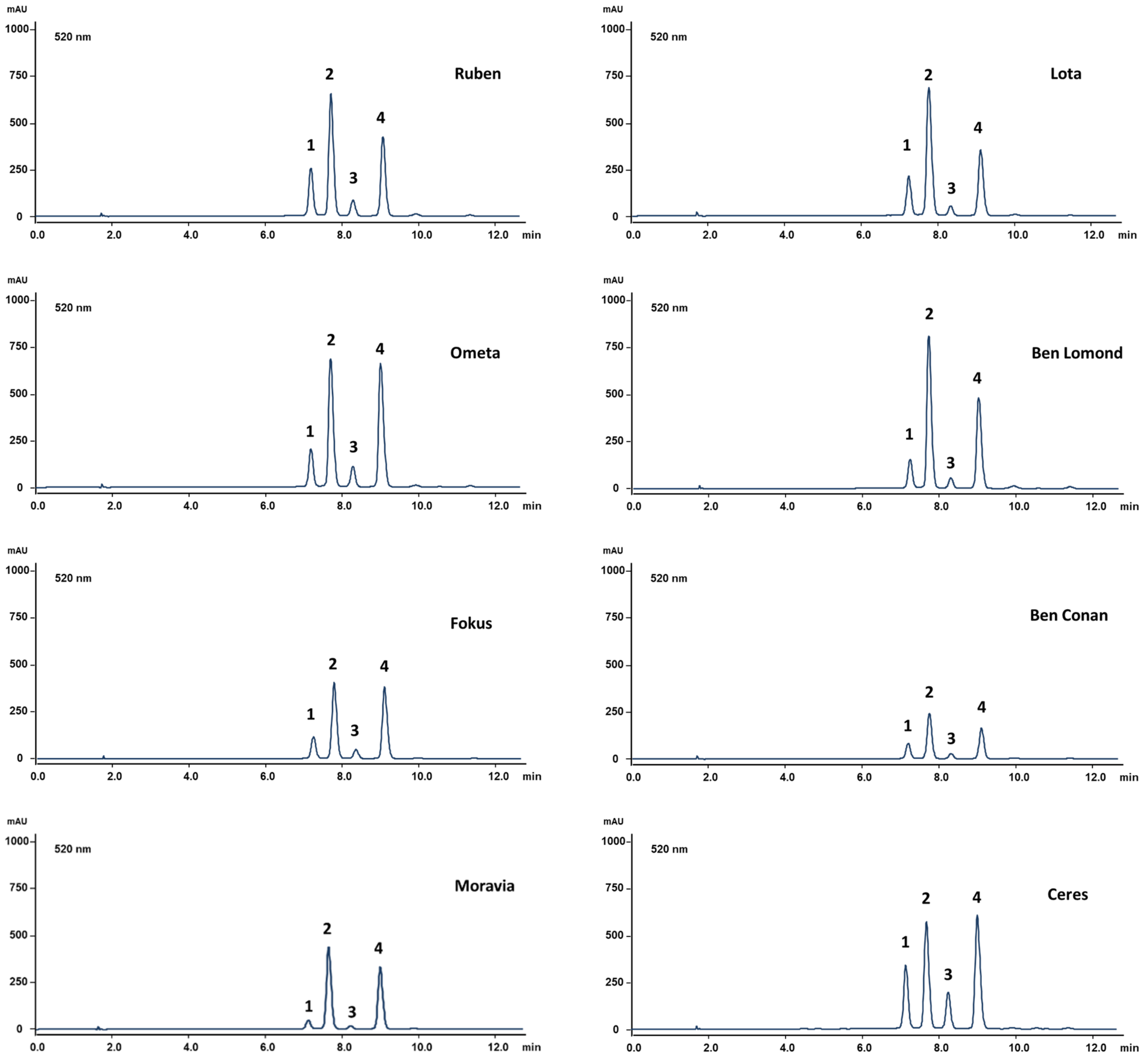

Figure S1: Chromatograms of anthocyanins content in several cultivars. (1) delphinidin-3-glucoside; (2) delphinidin-3-rutinoside; (3) cyanidin-3-glucoside; (4) cyanidin-3-rutinoside

2. Evaluation of anthocyanin content throughout the three-year experiment

Table S1: Total anthocyanin content in 15 blackcurrant cultivars in three years period

	Total anthocyanin content (mg/100 g)[a]			
**Blackcurrant cultivars**	**First year**	**Second year**	**Third year**	**Average**
Moravia	135.78	135.89	213.00	161.6 ± 44.6
Ometa	310.98	267.40	228.91	269.1 ± 41.1
Ben Conan	79.41	186.60	198.21	154.7 ± 65.5
Fokus	141.42	187.83	209.04	179.4 ± 34.6
Ceres	261.82	234.54	347.56	281.3 ± 59.0
Ben Lomond	217.38	130.95	282.66	210.3 ± 76.1
Lota	219.95	141.68	239.49	200.4 ± 51.8
Ruben	208.30	263.16	289.33	253.6 ± 41.4
Ben Hope	n.m.	211.20	301.89	256.6 ± 64.1
Ben Gairn	n.m.	235.17	353.59	294.4 ± 83.7
Consort	172.56	n.m.	n.m.	172.6
Tenah	202.02	n.m.	n.m.	202.0
Sejanec	186.27	n.m.	n.m.	186.3
Triton	n.m.	174.91	n.m.	174.9
Josta	n.m.	n.m.	93.48	93.5

[a] Calculated as a sum of four main individual anthocyanins

n.m. - not measured
